# More than just collateral damage. Ramifications of the pandemic for freedom of the press

**DOI:** 10.1007/s11616-021-00699-4

**Published:** 2021-11-29

**Authors:** Christina Holtz-Bacha

**Affiliations:** Lehrstuhl für Kommunikationswissenschaft, Friedrich-Alexander-Universität Erlangen-Nürnberg, Findelgasse 7–9, 90402 Nuremberg, Germany

**Keywords:** Freedom of the press, COVID-19, Access to information, Chilling effects, Disinformation, Press subsidies, Pressefreiheit, COVID-19, Zugang zu Informationen, Chilling-Effekte, Desinformation, Presseförderung

## Abstract

Confronted with a situation that was new to everyone, world leaders sought the best way to contain the pandemic caused by the coronavirus. The measures introduced, with lockdowns and curfews, affected fundamental rights such as freedom of movement and assembly, with implications for freedom of expression and freedom of the press. Demonstrations against the governments’ COVID-19 policies and the associated encroachments on citizens’ fundamental rights in turn led to attacks on media representatives and thus on the freedom of reporting. They were also further proof that attacks on the freedom of the media no longer come only from the state. The restrictions on journalistic work were by no means a surprise attack on the media, but the continuation of a worldwide trend that has been evident for several years, not only in authoritarian systems, but also in established democracies. For those who fear the free press and its watchdog function, the pandemic provided a welcome opportunity, under the pretext of public health, to tighten the reins a bit more and create facts that will endure beyond the pandemic. Financial support for the media, especially the printed press, to cushion the economic consequences of the pandemic and to secure newspapers for the future, establishes new dependencies that can affect free reporting in the long term.

## Abstract (de)

Konfrontiert mit einer Situation, die für alle neu war, suchten die Staats- und Regierungschefs der Welt nach dem besten Weg, um die durch das Coronavirus verursachte Pandemie einzudämmen. Mit Lockdowns und Ausgangssperren beeinträchtigten die eingeführten Maßnahmen Grundrechte wie Bewegungs- und Versammlungsfreiheit, was auch Auswirkungen auf die Meinungs- und Pressefreiheit hatte. Demonstrationen gegen die COVID-19-Politik der Regierungen und die damit verbundenen Eingriffe in die Grundrechte der Bürger*innen führten wiederum zu Angriffen auf Medienvertreter*innen und damit auf die Freiheit der Berichterstattung. Diese waren auch ein weiterer Beweis dafür, dass Angriffe auf die Medienfreiheit nicht mehr nur vom Staat ausgehen. Die Einschränkungen für das journalistische Arbeiten und Angriffe auf die Medien kamen keineswegs überraschend, sondern waren die Fortsetzung eines weltweiten Trends, der sich seit einigen Jahren nicht nur in autoritären Systemen, sondern auch in etablierten Demokratien zeigt. Für diejenigen, die die freie Presse und ihre Watchdog-Funktion fürchten, bot die Pandemie unter dem Vorwand des öffentlichen Wohls eine willkommene Gelegenheit, die Zügel etwas mehr zu straffen und Fakten zu schaffen, die über die Pandemie hinaus Bestand haben werden. Die finanzielle Unterstützung der Medien, insbesondere der gedruckten Presse, um die wirtschaftlichen Folgen der Pandemie abzufedern und Zeitungen für die Zukunft zu sichern, schafft neue Abhängigkeiten, die die freie Berichterstattung langfristig beeinträchtigen können.

## Introduction

The pandemic has drawn great attention to the role of the media in informing the population about the spread of the coronavirus and health policy measures, but also in dealing with disinformation, especially on social media. The political leadership in particular, in whose hands it is to find an answer to the pandemic, has an interest in finding support for their decisions and measures to overcome the crisis. Confronted with the unstoppable spread of the virus and the rapidly increasing number of infections, but also with uncertainty about effective measures to counter the pandemic, governments sought the backing of the news media to gain acceptance for their crisis management. In a situation that is new to everyone, media that remain true to their critical role, risk being accused of a lack of solidarity in the face of a national threat.

In addition to the consequences for people’s physical and mental health, as well as the financial challenges and the economic downturn, the pandemic found yet another victim in freedom of the press. Not only because they fear media criticism of their policies in dealing with the pandemic, governments around the world used the situation to put a curb on the media and keep them from performing their public responsibility. These interferences reached from denying journalists access to information, disinformation, different forms of censorship to closing down media outlets and physical threats. While public attention turned to the pandemic and, by putting health on top of the agenda, political leadership could rely on a general sense of community, it was not only leaders in authoritarian states who seized the opportunity to tighten the reins for the media, hinder their investigative research, thereby undermining their independence. Uncertainty about the development of the pandemic, the handling of an unprecedented situation, growing criticism of the measures to combat the virus, and impatience with their effectiveness made political leaders thin-skinned.

The consequences of the pandemic for the economy also entailed a further decline of the media’s advertising revenue and particularly that of the printed press, which exacerbated the already difficult situation of the newspaper market. Several countries therefore included the press in their support measures for the economy and increased or newly introduced press aids that may build up long-term dependencies.

## The role of the media in crises

The deterioration of its economic base hit the media at a time when their orientation function was in particular demand. Crises bring about a growing need for information among the citizens, which is especially directed at those who have decision-making power and are responsible for managing the crisis. The pandemic caused by the coronavirus developed into a crisis with certain peculiarities and can therefore only be compared to other types of crises to a limited extent.

Unlike natural disasters, which occur unexpectedly and rapidly and are usually geographically limited in their effects, the outbreak of the virus in China was surprising at the time, but not completely unforeseen due to warnings of the possibility of a pandemic in the past, e.g., by virologists or other people such as entrepreneur Bill Gates. The global spread of the virus unfolded within just a few weeks. Because virus outbreaks such as the avian flu or Ebola and even the SARS pandemic at the beginning of the century remained regionally restricted and, in the end, did not evolve into a global threat. The pandemic can therefore best be compared with environmental crises that are global in scale. In view of the earlier warnings and its dubious predictability, the pandemic could be described as a latent crisis; the infections finally made the crisis manifest (cf. Wolling 2016, p. 236).

The situation was new for everyone and therefore brought with it uncertainties on all sides. While science was researching the virus and the routes of infection, governments were looking for the right measures to fight the virus and slow its further spread. As long as there were no suitable medical means of containing the virus, restricting personal contacts to interrupt contagion routes was considered the best measure. However, confinements and restrictions on personal contact, and thus the determination to put public health first, constituted an interference with the personal freedoms of each individual, which are usually guaranteed in a country’s constitution. In democratic systems in particular, this led to controversies about the making of such decisions and their legitimacy because those measures were often enacted without the involvement of the legislative body.

The interruption of international travel, the hasty repatriation of citizens to their home countries and the closure of state borders made the handling of the pandemic a national issue that also exposed national egotisms. By calling the coronavirus “the China virus” once again in his short farewell speech on Joint Base Andrews before taking off for Florida and thus ascribing responsibility for the worldwide spread of the virus to China, U.S. President Trump tried to define the pandemic as the consequence of an attack from outside the country and thus a battle between “them” and “us”.

Such a situation of general uncertainty first and foremost challenges the information function of the media. In fact, as the Reuters Institute Digital News Report 2020 (cf. Newman et al. 2020, p. 10) confirmed, people worldwide turned to the news media to an increased extent and it was mainly the traditional media that were in demand. This was especially the case for television, which profited most from this trend. More specifically, as Casero-Ripollés for instance reported for the U.S., the audience’s need for information was reflected in an extraordinary increase in news consumption with the onset of the pandemic in March 2020, and this pertained to all age groups (cf. Casero-Ripollés 2020, p. 5). While traditional and social media enjoyed a growth of their audience, network and cable television garnered the highest percentages of frequent news consumption leading Casero-Ripollés to conclude that “Covid-19 has given relevance to television” (Casero-Ripollés 2020, p. 6). Surveys from the Nordic countries corroborated the findings from elsewhere that, when the crisis began, audiences turned preferentially to professional media and especially to television, including their online offerings, while the use of social media was less important (cf. Nordicom 2020).

To take another example, television news programs in Germany also saw their audience grow in 2020, with news on public service television in particular increasing its market share (cf. Haddad et al. 2021, p. 144). At the same time, trust in the media, which had suffered significantly in previous years, also rose considerably again in 2020. In particular, audiences placed great trust in the coverage of the pandemic; nearly two-thirds of adult respondents said in 2020 that they fully trusted the Corona reporting (cf. Jakobs et al. 2021, p. 155). The findings about the important role of public service television fit with the research presented by the European Broadcasting Union (2020, p. 7), which shows that the average daily viewing share of its members’ public service evening news in March 2020 was 20 percentage points higher than in the first quarter of 2019.

One year on, the Digital News Report 2021 (Newman et al. 2021) shows whether the findings from the early days of the pandemic hold or whether the increased interest in news was only short-lived. In general, the pandemic appears to have bolstered trust in the news. The report records an increase of six percentage points, bringing trust back to 2018 levels (cf. Newman et al. 2021, p. 9). In comparison, the U.S. features the lowest level of trust, which is likely due to the country’s strong political polarization. This divide is reflected in trust in news sources as well as in judgments about COVID-19 coverage (e.g., Gottfried 2021; Gottfried et al. 2020). Newman et al. (2021, p. 10) points out that the survey has not only documented an increase in the consumption of television news in several European countries, but has also shown people’s preference for reliable news sources in general. In those countries that feature strong and independent public service media, i.e., especially in Western Europe, trusted news brands can enjoy greater consumption, thus emphasizing the special role of this media sector which is also echoed in the success of the public service websites with the audience (cf. Newman et al. 2021, p. 11).

The results of a two-wave panel study from early in the pandemic (December 2019 and May/June 2020) by Van Aelst et al. (2021) complement the findings of the Digital News Report. This study involves 17 countries, but mainly refers to West European democracies. It shows an increase in news usage on television, online and in social media, while radio and newspaper usage declined. The researchers interpret the latter as a possible effect of the lockdown, which, for example, restricted the opportunities to buy the daily newspaper and listen to the radio on the way to work. This study also confirms the influence of trust in the news media and of concerns about the impact of Covid-19.

On the other side, as part of their crisis management political decision-makers are also dependent on the media for informing citizens about their policies, explaining the reasons for their decisions and thus lend them legitimacy, and for garnering support. Since the conditions for crisis communication have changed profoundly due to the development of the social network sites, which have proved their role as a fertile breeding ground for disinformation and conspiracy theories, the dependence of political actors on the journalistic media has grown.

How leaders across the world reacted to the spread of the virus, the measures they took and in which way they communicated them to the public, differed to a major extent. Some outright denied or tried to downplay the dangers of the virus—for pragmatic reasons, for example due to pressure from the business community or simply because of incompetence or to cover up uncertainties or avoid having to admit to wrong decisions. Others, who took the danger seriously and accepted the challenge, sought the solidarity of the media and relied on their support to convince the population of the need for tough measures that also restrict fundamental rights. However, criticism is considered dysfunctional and therefore inconvenient in such a situation.

As Dagenais (1992, p. 120) contends, major crises put the media in an “inescapable cycle” that always plays out the same way. At the beginning of a crisis, the media assume their basic democratic role by providing information that allows everyone to follow the course of the crisis and understand its implications. The second phase is a time of self-reflection, in which the media question their role in the crisis and discuss whether they have fallen victim to manipulations. In a third phase, experts from outside the media scrutinize the coverage of the crisis leading the media to acknowledge possible mistakes while they point at the same time to the crucial role they played in providing information. The cycle ends with a “double dialogue” (Dagenais 1992, p. 121) involving politicians and academics on the one hand, who analyze the role of the media in the crisis, and journalists on the other, who defend their handling of the situation.

In fact, the Reuters News Report 2020 concluded that overall news media have been rather supportive in the early stages of the pandemic (cf. Newman et al. 2020, p. 13). In crises that are perceived as a threat to the entire nation, citizens tend to rally around the flag and turn to those who have decision-making power and are thus believed to be the ones who can help overcome the crisis (e.g., Bækgaard et al. 2020). This effect also captures the media before they enter the phase of self-reflection and resume their watchdog role by scrutinizing the governments’ crisis management.

## Constraining the media

As dealing with the coronavirus outbreak jumped to the top of the public’s agenda and expectations were high for governments to contain the virus and thwart its economic and social consequences, public attention focused on the pandemic, while other issues took a back seat. It did not take long until first complaints about all kinds of interferences with freedom of the press came up. The encroachments were not only related to coverage of the pandemic, but reflected a general intolerance of criticism, suggesting that governments were taking advantage of the distraction to cut back on press freedom. Already in spring 2020, international organizations that focus on human rights and civil liberties published alarming assessments of how governments used the situation for cracking down on the media (e.g., Council of Europe 2020b, c; European Parliament 2020a, b). Not much later, on World Press Freedom Day on 3 May 2020, UN Secretary-General António Guterres highlighted the eminently important role of a free and independent press, especially in the pandemic, not least to counter the tide of disinformation, and UNESCO chief Audrey Azoulay ascertained that in the pandemic, as in war, truth was once again the first casualty (cf. UN News 2020).

Assaults on freedom of expression, which includes freedom of the press, were linked to restrictions on other fundamental communication rights, such as freedom of information and freedom of assembly. The infringements of press freedom did not occur suddenly with the onset of the pandemic, but rather fit into a trend that has been emerging for some time. For years, organizations such as Freedom House and Reporters Without Borders that regularly monitor the quality of civil liberties around the world, have bemoaned the decline of press freedom, not least in connection with the rise of right-wing populism (e.g., Freedom House 2019; see also Council of Europe 2020a). This has by no means only affected authoritarian regimes, but also established democracies, which should make the alarm bells ringing all the more.

In fact, as the Democracy Index drawn up annually by The Economist Intelligence Unit (2021, p. 6) summarized, citizens across the world in 2020 “experienced the biggest rollback of individual freedoms ever undertaken by governments during peacetime (and perhaps even in wartime)”. In Western Europe, formerly a stronghold of democracy, the index records the sharpest declines in scores for the categories civil liberties, which encompasses several variables indicating freedom of expression and the media, and functioning of government. At the same time, democratic backsliding continued in Central and Eastern Europe as well as in Latin America, and for the U.S. that the Democracy Index classifies as a flawed democracy, the report points to increasing threats to freedom of expression (cf. The Economist Intelligence Unit 2021, pp. 7–8).

With the onset of the pandemic and cornered by an unprecedented challenge, governments became creative to stop the spread of unpleasant news, criticism of their measures to get the pandemic under control, and doubts about their effectiveness. Their actions ranged from obfuscation, restrictions on access to information to sanctions for the spread of alleged disinformation. As soon became apparent, it was not only governments that were responsible for the interventions, but other actors entered the scene who also interfered with the work of journalists and thus impaired the freedom of reporting. The violations of press freedom became so blatant that, among other watchdog organizations, the International Press Institute (IPI) installed a COVID-19 Press Freedom Tracker for monitoring attacks on journalists and the press worldwide linked to the pandemic (https://ipi.media/covid19/?alert_type=0&language=0&years=0&country=0). Such government-imposed restrictions on journalistic work came along with the overall deterioration of information-gathering conditions for journalists through lockdowns, curfews, the shift of editorial work to the home office, financial and emotional distress, and the risk of contracting the virus themselves (cf. also Libert et al. 2021).

### Verbal or physical attacks on journalists

At the top of the list of press freedom violations recorded by the IPI are verbal or physical attacks on journalists.^1^ At first glance, it may come as a surprise that, compared with other regions of the world, the largest share in this category is accounted for by Europe with its large number of democracies in which the freedom of the media should be well protected. In fact, these findings demonstrate that it is no longer just the state that threatens press freedom, but that attacks on press freedom also come from other sides. This has been particularly evident with the surge of right-wing populism, which has discredited the traditional media as the “lying press” and “fake news” and called their credibility into question (cf. Holtz-Bacha 2020). With the persistence of the pandemic, multiple lockdowns and the concomitant restrictions on fundamental rights, criticism of government measures increased. Public demonstrations, which are based on the democratic freedom of assembly, were allowed under appropriate sanitary conditions in many countries even during lockdowns. Protest marches gathered Corona deniers and supporters of conspiracy theories and repeatedly resulted in verbal and physical attacks on those who wanted to cover the demonstrations and thus came into conflict with another fundamental right, which is likely to be reflected accordingly in the IPI statistics. Germany is a case in point. The country has a freedom of the press that is well anchored in the constitution and has been repeatedly strengthened through rulings by the Federal Constitutional Court. However, in addition to longtime efforts on the part of the state to implement surveillance regulations and data retention to combat crime, the media have come under considerable pressure in recent years from hostility and assaults by right-wing groups. Violent attacks on media workers during demonstrations against the government’s COVID-19 measures and other protests, mostly by the demonstrators, but also by the police were one reason for downgrading Germany from “good” to “satisfactory” in the Reporters Without Borders’ 2020 ranking (cf. RSF 2021a, b, p. 2). The Netherlands provide for another example in this context. The country regularly reaches one of the top places in the Reporters Without Borders and Freedom House indexes, but in 2021 it dropped one place from the previous year and now ranks sixth on the RSF index. Journalists were repeatedly attacked when trying to cover protests against the government’s lockdown policy. The public broadcaster NOS became a particular target for the protesters. In order not to endanger their journalists, NOS even temporarily removed the logo from their broadcast vehicles (cf. DW 2021). Similar incidents of violent attacks against journalists also occurred in Italy (cf. RSF 2020d), a country that has fallen further and further down the press freedom rankings over the years and is 41st on the RSF 2021 index. The example of Italy also shows how anti-media sentiment builds up when politicians insult and condescend to the media, thus offering justification for threats against the media and verbal and physical attacks on journalists.

### Arrests and criminal investigations

Arrests and criminal investigations rank second among the different kinds of assaults on freedom of the press assessed by the IPI. Most of these incidents are recorded for the Asia-Pacific region, with Africa a distant second, closely followed by Europe. The first place in this category for the Asia-Pacific region is not surprising, as this area includes China, a country that is one of the notorious predators of press freedom and takes rigorous action against inconvenient journalists. This region also includes India, the world’s largest democracy, for which Freedom House recorded “alarming departures from democratic norms” over the last years (Repucci 2020, p. 2). This also includes setbacks for freedom of expression; India now ranks 142nd on the RSF Index. With the onset of the pandemic, Prime Minister Narendra Modi tried to influence the media by asking for positive coverage of his COVID-19 policies. When the government requested the Indian Supreme Court to compel the news media to disseminate information only after clearance by the government, the court refused to do so but instructed the news media to use the official announcements in its COVID-19 coverage (cf. Rodrigues and Xu 2020, p. 128). Criticism of the government’s response to the pandemic was pursued with criminal investigations and arrests.

In Europe, Serbia that has been negotiating its accession to the EU for several years stood out in this category. On the RSF Index, Serbia ranks 93rd just behind Hungary. Not least because of the harassment of journalists, Freedom House also gives Serbia poor marks in its Freedom in the World 2020 report (cf. Freedom House 2020, pp. 14, 24). During the pandemic, Serbia used various measures to try to control the media and prevent criticism. Journalists were threatened, there were arrests and indictments (cf. Cendic and Gosztonyi 2020, pp. 23–24; International Press Institute 2020b).

### Restricting and denying access to information

Restrictions on access to information that the IPI tracker lists in third place among press freedom violations in the pandemic have always been a convenient means to keep the outcome of political decisions and measures in the dark and control media reporting. Even in established democracies, there have been tendencies for some time to selectively handle media access to information, for example at party conventions or press conferences, and to exclude undesirable journalists. Although it is recommended for crisis management to inform the public and also admit any uncertainties to avoid loss of credibility and trust, many governments restricted access to information during the Corona crisis in order to influence the media’s construction of the pandemic, the evaluation of mitigation measures, and their general acceptance. In this way, critics can be shut out, statements can be kept vague, and bothering inquiries can be avoided. However, restrictions on access to information come in various disguises, are not always obvious and therefore difficult to pin down. They not only apply to official information provided by state authorities but also by other organizations and institutions that are involved in fighting the pandemic. That is also why the cases that fall under this category in the IPI tracker are likely to contain a significant underestimation.

In addition to the deliberate concealment of actions in the crisis management and of the development of the pandemic, many employees of relevant institutions were working from their home and therefore did not always have access themselves to records needed to answer (media) inquiries and meet the requirements of freedom of information regulation.

In the category “Restrictions on access to information,” the Asia-Pacific region, which includes many authoritarian regimes where access to information is constricted anyway, again takes first place, followed at some distance by Europe. However, the methods used to impede or deny journalists access to information differ significantly. About 88% of the cases in the Asia-Pacific region involved the revocation or denial of press cards or accreditation, and about 10% involved travel restrictions or bans. In Europe, on the other hand, limiting access to officials and press conferences topped the list with about half of the cases. Almost 30% of the cases related to the repeal of Freedom of Information Acts or the extension of deadlines within which requests must be answered; the denial of a press card accounted for about one fifth.

In the United Kingdom, for instance, the country where press freedom began but which has slipped in ranking over the years and is currently 33rdon the RSF index, the prime minister seemed to take a cue from the anti-press stance of then U.S. president Trump. Faced with criticism of his initial handling of the pandemic, Boris Johnson stepped up his bashing of the press; inconvenient news media were vilified as “campaigning media” and hindered in their work. Restrictions were placed on daily press briefings. Journalists were relegated to virtual participation; their possibilities to ask questions were limited. Foreign correspondents were excluded from press briefings altogether (cf. RSF 2020c). In Spain, to give another example, journalists were also not able to ask questions in government press conferences in the early weeks of the pandemic. They had to hand in their requests in advance and in writing to the State Secretary for Communication, who was supposed to forward them to the responsible ministers but seemed to filter the questions. Only after pressure by journalists and journalists’ associations the government gave in and allowed direct interaction during press conferences again (cf. RSF 2020b). In Austria, where the adoption of a freedom of information act has long been envisaged but has still not been realized, there have been various complaints about the difficulties of obtaining information at all or detailed pandemic-related information. This also applied to the situation in the popular ski resort of Ischgl, which became a hotspot due to a high number of infections in the early days of the pandemic (cf. Vogt 2020).

The lines between restrictions on access to information and lack of transparency regarding pandemic containment measures and their effectiveness are blurred, which should also account for an underestimation of cases attributable to this category. In Israel, for instance, then prime minister Netanyahu adopted a centralistic approach to managing the crisis, facilitated by a law passed early in the first wave of the pandemic allowing him to make decisions without seeking approval by the parliament (cf. Gesser-Edelsburg and Hijazi 2020). Netanyahu’s crisis management combined an intimidation strategy towards the public with a public relations strategy in the interest of his image, thus interweaving health and political matters. His information policy was characterized by a lack of transparency; basic facts were only accessible to a small circle of people directly involved in crisis management (cf. Gesser-Edelsburg and Hijazi 2020, p. 2995).

Emphasizing that in a public health crisis it is particularly important to ensure an informed citizenry, as early as March 2020, the Reporters Committee for Freedom of the Press (RCFP) in the U.S. therefore issued guidelines for newsgathering under the conditions of the pandemic and called upon authorities to ensure processing of requests for information related to COVID-19 (cf. RCFP 2020). This appeal came at the same time as the Washington D.C. City Council, for example, approved the COVID-19 Response Emergency Amendment Act of 2020, which would allow government agencies to delay answering records requests in relation with the pandemic (cf. RCFP 2020).

### Censorship

According to the IPI tracker and with some distance to other forms of media freedom violations, censorship ranks fourth. The censorship category includes publication bans, take-down orders and forced deletion, which are infringements accounting for about 80% of the cases, forced closure of media outlet and the blocking of a website. Here, Europe ranks first, with publication bans and take-down orders even accounting for more than 93% of the cases. This type of intervention also dominates in the Asia-Pacific region and the Americas, while in Africa and the MENA region censorship was mainly exercised through forced closure of media outlets.

In Hungary, together with Poland the EU’s black sheep not only when it comes to media policy and freedom of the press, the government took advantage of the pandemic and closed down Klubrádió, which was the last remaining independent radio broadcaster in a media landscape that has been under siege for years by the government of Victor Orbán. In Malta, which, as the Council of Europe’s Committee on Legal Affairs and Human Rights (2019) noted in connection with the investigation of the murder of the investigative journalist Daphne Caruana Galizia, manifests some “fundamental weaknesses in [its] system of checks and balances” (Council of Europe 2019, p. 5), journalists complained about their questions regarding the government’s COVID-19 measures having been censored on TVM, the country’s public broadcasting service. As the Maltese Journalists’ Association (IGM) criticized, the broadcaster’s interventions were based on a directive on COVID-19 reporting issued by the Broadcasting Authority (cf. Aquilina 2020; IGM 2020).

### Fake news regulation and chilling effects

Without any doubt, the pandemic gave rise to a flood of disinformation and conspiracy theories. The coronavirus brought along another virus. Together with the health risk, a “pandemic of disinformation” unfolded (Tagliabue et al. 2020), “infodemic” became the new buzz word. Misleading claims by self-appointed experts hindered the enforcement of health-related measures and also sparked protests in many countries compelling authorities to fight on two fronts, against the coronavirus and against false information. The latter, however, was also utilized for silencing inconvenient criticism from the news media. With 17 cases scattered across all regions except Africa, excessive fake news regulation ranks fifth among the media freedom violations recorded by the IPI Tracker. These regulations are used against allegedly false information and serve to justify actions against media companies or individual journalists. The interpretation of what is considered fake news is left to the authorities, who can declare any deviation from official statements or criticism of government measures to be fake news and punish the media accordingly. Fake news regulation often came along as part of wider emergency legislation that governments released to procure extraordinary decision-making opportunities in the pandemic, allowing them to bypass the usual legislative route. It is this context that leads Papadopoulou and Maniou (2021, p. 18) to declare that digital journalism was the main target of the restrictive measures for the media and that the pandemic was a welcome opportunity to tighten its reins.

IPI lists the countries that passed regulations criminalizing the dissemination of fake news related to the pandemic. According to the EIU Democracy Index 2020 (The Economist Intelligence Unit 2021) many of those countries feature authoritarian systems, others are counted among hybrid systems or flawed democracies and several of them are notorious predators of freedom of the press such as the Central Asian states, Russia and Vietnam. Authoritarian regimes that gag their media at all times did not necessarily need new tools for steering news dissemination during the pandemic because they are kept in line one way or another anyway. If they enacted new rules nevertheless and especially for getting a grip on “fake news” during the pandemic, those served as a kind of fig leaf allowing the authorities to control COVID-19 related information specifically under the pretext of the health crisis and for dispelling insecurity among the population. In many cases, allegations of spreading fake news or the degradation of media as fake news or lying press are likely to be responsible for the attacks on journalists and the media, as the IPI Tracker records as violations of press freedom. Fake news regulation provided a convenient basis for the prosecution of journalists through arrests, civil lawsuits and criminal investigations.

EU member states Hungary and Romania as well as EU applicant Bosnia-Herzegovina are among the countries that passed fake news regulation during the pandemic. The Hungarian prime minister Victor Orbán, who has been pursuing an illiberal media policy for years (cf. Polyak 2019, p. 297), obtained extensive powers to govern by decree based on an emergency law passed in March 2020 right at the beginning of the coronavirus outbreak. This allowed him to enforce the criminalization of the spread of disinformation, which became punishable by fines and up to five years in prison (cf. OSCE 2020). The state of emergency ended in June but left the government with the right to rule by decree which was used to reintroduce the state of emergency again in November 2020.

Bulgaria had similar plans. RSF ranks media freedom in the country at 112 which puts worst among all EU member states. The parliament passed a law that would criminalize the dissemination of COVID-19-related disinformation (cf. RSF 2020a). It provided for prison sentences of up to three years and a fine of about 5000 euros. However, with regard to the impact on freedom of expression the Bulgarian president vetoed the law, preventing it from taking effect (cf. European Commission 2020d, p. 17). Neighboring Romania that appears on rank 48 on the RSF Press Freedom Index declared the state of emergency in mid-March 2020 by a presidential decree that restricted some fundamental rights until May. The decree also included measures to fight the distribution of what authorities deemed to be fake news, allowing for the removal of reports or completely closing down websites without any right of appeal for the affected media outlets (cf. RSF 2020a). Among the “multiple misdeeds […] in the response to the COVID-19 pandemic” (Thomson and Ip 2020, p. 16), recorded in Bosnia-Herzegovina, was a decree issued early in the pandemic for the autonomous entity Republika Srpska, which criminalized the dissemination of misinformation or reports that could cause panic, disturb public order or peace and could lead to substantial penalties. The decree was dismissed again in April 2020.

While governments sought to contain pandemic and infodemic, and some of them exploited the situation to influence COVID-19 reporting and suppress critical voices, journalists from 125 countries identified political leaders and elected officials (46%) as the top source of disinformation in a mid-2020 survey right behind regular citizens (49%) (cf. Posetti et al. 2020, p. 14). The dissemination of false information was also attributed to identifiable government agencies or their spokespeople (25%) and government-sponsored troll networks (23%). Another 34% of the respondents felt that propagandistic or heavily partisan media and state-owned media contributed to the infodemic. These data cover 125 countries with different regimes, and they are a snapshot from an early phase of the pandemic. Together with the measures governments adopted to control the flow of information, they nevertheless demonstrate very well the struggle for the power of definition in dealing with the virus.

Beyond official regulations, decrees and laws released to influence the pandemic-related public discourse fake news regulations, particularly where they criminalize the dissemination of whatever is deemed false information, entail chilling effects that influence and hinder the media and the work of each individual journalist. But the climate for the media is also determined by aggressive rhetoric toward them, as cultivated above all by authoritarian and populist leaders around the world, undermining general trust in the media and their credibility. In the key findings of its 2019 report on freedom of the media, Freedom House explicitly pointed to populism as one reason for the decline of press freedom even in established democracies (cf. Freedom House 2019, p. 1). And the Reuters News Report 2020 (cf. Newman et al. 2020, p. 14) showed how trust in the news has eroded globally. In addition to anti-media rhetoric by political leaders, harassment and defamation campaigns through social media and during public protests contribute to the intimidation of journalists. In its 2020 Rule of Law Report, the European Commission pointed out that chilling effects due to strategic lawsuits for the purpose of intimidation and financial damage (SLAPP) as well as threats, attacks and smear campaigns against journalists, as they occur in several member states, “entail the risk of a shrinking public debate on controversial societal issues” (European Commission 2020b, p. 20). In a case study, Cendic and Gosztonyi (2020) detailed the chilling effects on journalists in Hungary and Serbia and thus two countries that quickly exploited the pandemic to further restrict freedom of the media. Slovenia can also serve as an example here. In this country, which ranks 36th on the RSF index, SLAPP lawsuits are used to “bleed the journalist and outlet of money, energy and other resources until they stop reporting on a story. Or to stop watchdog journalism altogether.” (International Press Institute 2020a).

The overall deteriorating climate for the media has made the safety of journalists a burning issue as is for instance indicated by “Platform to promote the protection of journalism and safety of journalists” established by the Council of Europe in 2014, and, more directly related to the pandemic, the resolution on “Threats to media freedom and journalists’ security in Europe” adopted by the Council of Europe’s General Assembly in early 2020 (cf. Council of Europe 2020c) or the COVID-19 guidelines for courts and judges proposed by the Unesco (2020) for the purpose of protecting and promoting freedom of expression. All of this adds up to intimidation, chilling effects and self-censorship for journalism that are not easily captured by the annual media freedom indices because they are not always manifest.

## Further weakening of the press through economic pressures

With the onset of the pandemic the printed press found itself in a paradoxical situation. Although the demand for information increased significantly in the crisis, which particularly benefited the traditional news media, the economic pressure on newspapers intensified because advertising revenues plummeted as a consequence of the economic downturn. This hit a media sector that had already suffered considerable losses in readership and advertising income in recent decades. Because of the special function of newspapers for the formation of political opinions and decision-making, and in order to ensure pluralism in the newspaper market, some European countries have supported their newspapers with state aids since the 1970s. However, the support schemes differ considerably. They comprise direct and indirect support reaching from subsidies for distribution and production to tax breaks to journalism education. Such aids are controversial not only because their effectiveness is difficult to prove, but also because of the danger of making newspapers dependent on the state while covering structural deficits. It is difficult to establish non-discriminatory selection criteria for the aids that relate solely to economic variables and not in any way to content, so as not to be suspected of interfering with freedom of the press. In addition, press aids are a delicate matter because state subsidies are generally prohibited under EU competition law and only approved by the European Commission under specific conditions. Due to the aggravated situation of the press in the pandemic, several states debated to temporarily increase or introduce press aids.

Sweden, for instance, together with other Nordic countries a pioneer of press subsidies, introduced a temporary aid measure to complement the existing schemes of media support and compensate newspapers for the significant losses in advertising revenues due to the COVID-19 pandemic. Further support was intended by making advance payment of press support possible and by removing the requirement that 55% of editorial material must be produced in-house (cf. Ministry of Culture 2020). In the spring of 2020, the Danish government established a similar temporary aid measure for print and digital news media, periodicals and magazines, and commercial radio stations. With reference to the particularly large decline in advertising revenues during the pandemic, Denmark also launched an aid program for weekly local newspapers in spring 2021 (cf. European Commission 2021a).

Austria, which features a press aid scheme that is contentious because it more or less follows a shotgun principle, granted its newspapers special support of more than 12 million Euros already in April 2020. Because the amount of funding is based on circulation, and newspapers with a high circulation therefore also receive higher funding, the tabloid *Kronen Zeitung*, which is close to former Chancellor Sebastian Kurz’s ruling Austrian People’s Party (ÖVP), once again pocketed the largest chunk. The fact that the quality newspapers got much smaller amounts rekindled the dispute about the Austrian press subsidy system. The same applies to the allocation of state advertising, which contributes substantially to the press subsidies. The fact that “there are no rules ensuring a fair distribution of state advertising among media outlets” was even critically highlighted in the European Commission’s Rule of Law Report on Austria (cf. European Commission 2020c, p. 11).

In the summer of 2020, France, which probably has the most diverse press support system in Europe, adopted a comprehensive two-year program that combines aid for the pandemic-stricken press with support for maintaining pluralism in the newspaper market and measures for an ecological and digital transition. In a first step, the French parliament pledged 106 million in emergency aid, and President Emmanuel Macron, who is up for re-election in 2022, contributed another 377 million to support the press in the next two years (cf. Gouvernement 2020). With its extensive system of press subsidies, France is also an example of how press subsidies are not always successful in overcoming structural weaknesses of the market and how newspapers thus become permanently dependent on state support. In a country where retail sales have traditionally been much more important than subscriptions, newspaper distribution is such an enduring problem. Therefore, one focus of the French aid package was newspaper distribution, which included 80 million euros for the relaunch of the press distribution company, an increase in the subsidy for newspaper sellers and tax credits for newspaper subscribers.

Even Switzerland, which has always refused to subsidize the press to preserve their independence and only supports delivery by mail, finally agreed to temporary emergency aid (cf. Bundesamt für Kommunikation 2020). Initially limited to six months, then extended again until mid-2021, the government offered free delivery of those subscribed daily and weekly regional and local newspapers that are eligible for a reduced delivery rate under the usual regime of indirect funding. In addition, the government granted a delivery discount for those newspapers (with higher circulation) that do not qualify for the indirect funding at other times.

In Germany, on the other hand, which, like Switzerland, has always been reluctant to support the press because of the risk of interference with press freedom, the plan to support the printed press failed. In the summer of 2020, the Federal Government announced an aid package of 220 million euros aimed at subscription newspapers and magazines as well as advertising papers and intended to serve their digital transformation. This package was heavily criticized, not least by publishers, who did not consider the conditions for applications to be practicable, but also because they feared an end to the printed press with the promotion of further digitization (cf. Lipinski 2020; Niemeier 2020). With the announcement of this support, a plan from the previous year was off the table, which proposed a subsidy for newspaper delivery after costs had increased due to a rise of the minimum wage that also applies to deliverers. This measure was also seen by publishers as a move away from the printed press. In April 2021, the Ministry of Economics finally signalized that it would not pursue the idea of a press subsidy any further thus leaving newspapers in a difficult situation after the pandemic-related decline in advertising revenues (cf. Tieschky 2021).

In Britain, where there is also no press subsidy, the Minister of Culture resorted to a very unique way of supporting newspapers, many of which had to lay off numerous journalists due to the decline of advertising revenue. Although the minister identified the press as the “fourth emergency service,” he limited the support for the press in the pandemic to an appeal to citizens to buy newspapers. And he also called on advertisers to refrain from blacklisting during the crisis and to accept their ads in the online editions to appear next to COVID-19 related articles (cf. Drury 2020).

Finally, Romania serves as an example of how such funding can be used selectively to favor certain media outlets sympathetic to the government. During the pandemic, the government announced financial support of 40 million euros, which was not distributed according to need, but strengthened clientelist networks (cf. Csaky 2021, p. 15).

In December 2020, the EU pledged financial support for the news media and for its digitization with an “Action Plan to Support Recovery and Transformation” (cf. European Commission 2020a). However, this project focuses on the audiovisual industry and largely leaves out the printed press, which, however, is in line with the European Commission’s competency in the media sector. Not least in connection with the pandemic, the Commission has finally become aware that not only economic problems threaten freedom of the media in the member states, and now also wants to devote itself to strengthening media freedom and pluralism in Europe and preparing a “European Media Freedom Act” (cf. European Commission 2021b).

## The media—doomed to long COVID

In its 2020 Rule of Law Report, the European Commission called the COVID-19 pandemic a “stress test for rule of law resilience” (European Commission 2020b, p. 6). The maxim of putting health first inevitably led to the restriction of individual fundamental rights. This also applies to freedom of the press. The health-threatening pandemic came hand in hand with a “constitutional pandemic” (Thomson and Ip 2020, p. 5). Although political leaders depend on the mediating function of the media for finding support for their decisions and in crisis situations there is also increased demand for news by citizens, it was by no means only authoritarian states that reacted with restrictions on journalism with the onset of the pandemic. In some cases, the restrictions were due to uncertainty about how to deal with the new virus, but in other cases induced by cool calculation and the exploitation of the crisis situation while attention was diverted and priorities were focused on combating the pandemic.

Many governments feared the critical discussion of their response to the pandemic and therefore tried with various measures to make it difficult for the media to exercise their watchdog role. Such measures ranged from lack of transparency and restrictions on access to information to censorship and criminalization of alleged misinformation, taking chilling effects and self-censorship into account. In addition, state and state-dependent institutions themselves contributed to the flood of misinformation that accompanied the pandemic. At the same time, however, it became apparent that threats to press freedom no longer emanate solely from the state. Protests against the restrictions of fundamental rights repeatedly led to attacks on media workers by demonstrators, who thus themselves violated the right to freedom of the press.

The encroachments on free reporting that accompanied the pandemic worldwide have once again shown that press freedom can never be considered secure, and is vulnerable even in established democracies. The multitude of admonitions and warnings published by international organizations soon after the coronavirus outbreak and the 2020 reports of those organizations that regularly measure the quality of press freedom in the world and register violations gave testimony to the threat that press freedom faces from the pandemic. The violations of press freedom regularly registered by the international watchdog institutions document that impediments to reporting have become a daily occurrence also in Western European countries, which have long been a haven of press freedom. Under the cover of the pandemic, the already creeping erosion of this fundamental democratic right intensified. The cases mentioned here are only examples of this development and they are by no means exhaustive.

The dramatic decline in advertising revenue for newspapers has worsened the economic situation of this media sector and brought about austerity measures that weaken the media in carrying out their role in democracy. Temporary and extended aid schemes, as introduced by many countries, further increased dependence on state support and created new liabilities. The consequences of these measures and other interventions in the media market will not disappear when the pandemic subsides, so that long COVID phenomena can also be expected in the media sector. Considering the focus of many support measures for newspapers on digitization, it does not come as a surprise that the Reuters Digital News Report 2021 calls the “coronavirus another “nail in the coffin” for print” (Newman et al. 2021, p. 13).

For years, press freedom has been deteriorating worldwide. It is no longer just the notorious rascals and authoritarian regimes who fear a free press, but increasingly also democratically governed countries in which the media are coming under pressure in one way or another. Against this background, the interference with freedom of the press that we have witnessed since the onset of the pandemic and under its guise is more than just collateral damage, but an indicator of the vulnerability of democracy.

## Footnotes

1 References to the data of the IPI COVID-19 Tracker reflect the situation in May 2021 (https://ipi.media/covid-19-tracker-in-graphics/).
